# Prevalence of Duodenal Diverticulum in South Indians: A Cadaveric Study

**DOI:** 10.5402/2013/767403

**Published:** 2013-11-07

**Authors:** Sulochana Sakthivel, Kavitha Kannaiyan, Sivakami Thiagarajan

**Affiliations:** ^1^Aarupadai Veedu Medical College, Pondicherry 607402, India; ^2^Department of Anatomy, Thanjavur Medical College, Thanjavur 613004, India

## Abstract

*Background*. Duodenum is the second most common site of diverticula after the colon. Diagnosis of duodenal diverticula is incidental and found during other therapeutic procedures. In 90% of cases, they are asymptomatic, and less than 10% develop clinical symptoms. The difficulty to ascertain the true incidence of duodenal diverticula demanded for the present study to elucidate the prevalence of the duodenal diverticulum in South Indians. *Materials and Methods*. One hundred and twenty specimens of duodenum were utilized for the study. The prevalence, anatomical location, and dimension of duodenal diverticulum were studied. *Results*. Among the 120 specimens of duodenum, five specimens had solitary, extraluminal, and globular-shaped diverticula in the medial wall of the duodenum. In three (60%) cases, it was found in the second part of duodenum and in two (40%) cases in the third part. The mean size of the diverticula was 1.4 cm. *Conclusion*. In the present study in South Indian people, the prevalence (4.2%) of duodenal diverticula is low comparable to other studies in the literature. Even though most of the duodenal diverticula are asymptomatic, the knowledge about its frequency and location is of great importance to prevent complications like diverticulitis, hemorrhage, obstructive jaundice, and perforation.

## 1. Introduction 

Duodenal diverticulum was first reported by a French pathologist Chomel in 1710 [1] and was diagnosed radiologically by Case [2] in 1913. Duodenum is the second most common site of diverticula in alimentary tract after colon, followed by jejunum, ileum, and stomach [3, 4]. Although associated with complications like diverticulitis, perforation, obstruction, or haemorrhage, the majority of duodenal diverticula are asymptomatic [5, 6], more often coming as a surprise on gastrointestinal series. 

Duodenal diverticula are found in 0.16 to 6% of upper gastrointestinal barium series and up to 23% of endoscopic retrograde cholangiopancreaticographies [6, 7]. However, reported incidence from cadaveric studies could be as high as 31.8% [8]. In recent review of the literature, there were a few cadaveric studies on the incidence of duodenal diverticulum throughout the world, and none from South India. To the best of our knowledge, the present study should be the first to highlight the prevalence and anatomical location of duodenal diverticula in South Indians.

## 2. Materials and Methods

One hundred and twenty specimens of duodenum from 108 male and 12 female subjects, in the age group of 25 to 63 years, procured from the Department of Anatomy, Thanjavur medical college, Thanjavur, and Department of Anatomy, Aarupadai veedu medical college, Puducherry, were utilized for this study, between the years 2005 and 2010. The specimens of duodenum and pancreas en block were removed, serially numbered, and preserved in 10% formalin. Specimens with duodenal diverticula were identified and carefully dissected, and its anatomical location on the duodenal wall was studied. Dimensions of the diverticula were measured with a caliper. The results obtained were compared with those reported previously. 

## 3. Results

Of the 120 specimens, diverticula were found in five specimens, the prevalence being 4.2%, and were located on the medial wall, which is morphologically the mesenteric border of the duodenum. They were solitary, globular-shaped, and extraluminal with the fundus of the diverticula posterior to the duodenum, partially buried in the pancreas. Of the five specimens with diverticula, three (60%) were present in the second part and two (40%) in the third part of the duodenum. 

**Figure 1:**
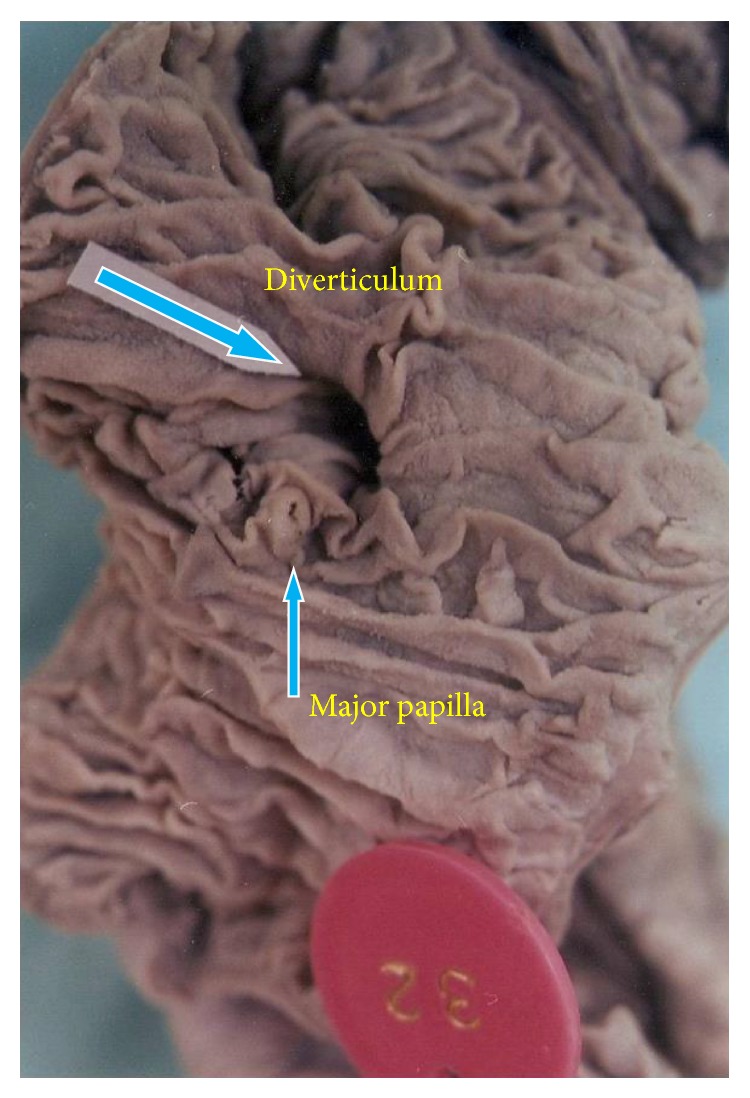
Duodenal diverticulum in the second part.

**Figure 2:**
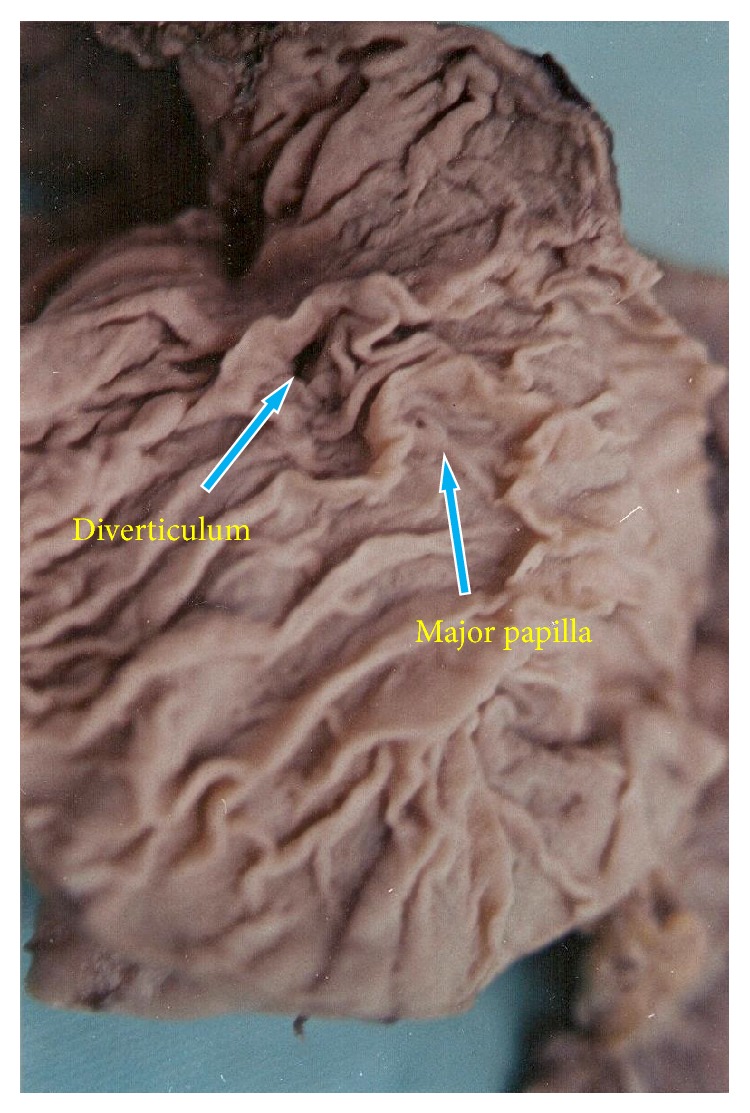
Duodenal diverticulum in the second part.

**Figure 3:**
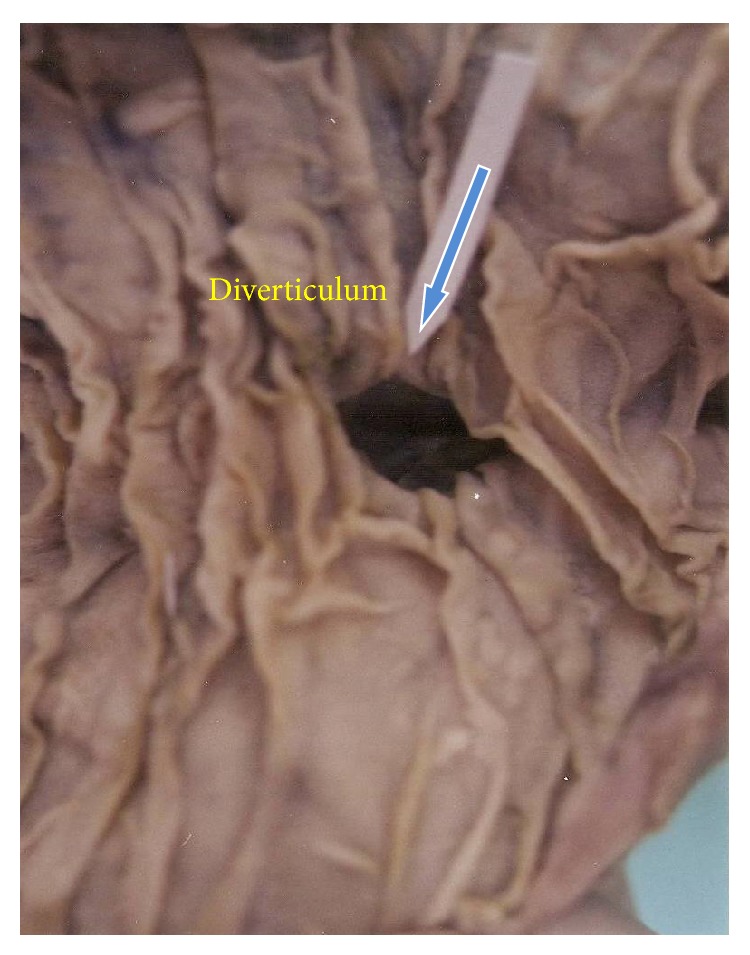
Duodenal diverticulum in the third part.

Diverticula in the second part of duodenum were located in the periampullary region, above the major duodenal papilla. No diverticula were found in the first or the fourth part. Sizes of the diverticula in the second part (Figures 1 and 2) were 2.5, 0.7 and 0.5 cm and in the third part (Figure 3) 3 cm and 0.5 cm. (mean—1.4 cm). In the present study, diverticula were found only in the male subjects.

## 4. Discussion

Duodenal diverticula can be classified into intraluminal duodenal diverticulum (IDD) and extraluminal duodenal diverticulum (EDD). IDD are congenital, resulting from defective recanalisation of duodenal lumen during fetal development [9] with coexistent congenital anomalies [10]. EDD are acquired or false diverticula, resulting from mucosal herniation at the point where blood vessels penetrate the intestinal wall, which also explains their typical location at the medial or pancreatic border [11], in 88% of cases. Only 4% of them occur on the lateral wall of the duodenum [12]. In the present study also, the diverticula were extraluminal and found on the medial wall, which is morphologically the mesenteric border of the duodenum. 

Even though some studies state that there is no gender predisposition [4, 5, 13], Grant Boileau [14] came across two diverticula in 11 female subjects as compared to 13 from 122 male subjects. In Case's [2] series of 85 cases of duodenal diverticula, 60% occurred in females. Mackenzie et al. [15] have also reported a female preponderance in the ratio from 1.6 to 1. However, in the present study, diverticula were found only in the male subjects, and the absence of the diverticula in female subjects could be due to less study population, which was also one of the limitations of our study. 

Review of the literature demonstrates that the incidence of duodenal diverticulum is highly variable according to the diagnostic procedure used. The incidence in upper barium series is 0.16 to 6% and in ERCP studies 09–23% [5–7]. A study in Mexico by Acuña et al. [16] reported an incidence up to 11.6% by endoscopic cholangiography. In autopsy series by Akhrass et al. [17], an incidence of 06–22% has been reported. In a study done in 105 specimens, 14 cases (13%) of duodenal diverticula with 13 solitary and one multiple were reported by Baldwin [18] in 1911. Of the 133 cadavers examined by Grant Boileau [14], diverticula were found in 15 subjects (11.2%), with 11 solitary and four multiple. Among them, 12 were globular in shape, three were conical, and five were tubular. Ackermann [19], in an anatomical study of 50 cadavers, reported an incidence of 22%, with eight solitary and three multiple diverticula. The highest on record to the present date is 31.8%, in a cadaveric study by Minoru and Atsuyoshi [8]. Thus, incidence of duodenal diverticula from various cadaveric studies is certainly higher than the incidence from any other diagnostic procedure, in view of the fact that the search for diverticula is more accurate in cadavers than visualizing them in any other methods of investigations. However, the present study revealed a lower prevalence of 4.2% when compared to that in the literature (Table 1). 

Most of the duodenal diverticula occur in the periampullary region of the duodenum, within 2.0 cm of the ampulla of Vater [17, 20, 21]. Several studies confirmed that the second part is the most common site followed by the third and fourth part of the duodenum. In a study by Lapin et al. [22], 62% of duodenal diverticula occurred in the second part, followed by the third (30%) and fourth part (08%). Scudamore et al. [23], in their study, showed that 82% of duodenal diverticula occurred in second part, 10% in the third, and 08% in the fourth part. In the present study as well, three diverticula (60%) were found in the second part, in the periampullary region, above the major duodenal papilla, and two (40%) in the third part of the duodenum, of the total five diverticula. Distribution of duodenal diverticula in various parts in duodenum is shown in Table 2. 

Despite the fact that duodenal diverticula are asymptomatic in 90% of cases, the higher frequency in the second and third part is associated with increased incidence of biliary stones, pancreatitis, and biliary and pancreatic anomalies. Concurrently, size of the diverticula is also of clinical importance since jaundice, cholangitis, and obstruction of pancreatic duct or bile duct have been reported due to the pressure effects [24]. In Case's radiological study of 85 cases, the average size of the diverticulum was 2.8 cm. In the present study, the size of the diverticula was 0.5 to 3 cm (mean: 1.4 cm), as compared to the mean value of 1.7 cm (range: 0.4 cm to 4.5 cm) by Wiesner et al. [25]. In comparison with other studies, the size of the diverticula was also smaller in South Indians. However, the dimensions of the diverticula in cadavers may be smaller than in life because of shrinkage during the embalming process [19], which is a disadvantage in our study. 

Since less than 10% of patients develop nonspecific clinical symptoms like abdominal pain or discomfort [5], diagnosis of duodenal diverticula is incidental, found only during other diagnostic or therapeutic procedures. However, 6.5% of patients may develop complications [27]. In the most common complications, being hemorrhage and pancreaticobiliary diseases, the clinical presentation may mimic acute cholecystitis, acute pancreatitis, or peptic ulcer disease [3, 7, 28]. Even though complications of diverticula are recognized with the advent of CT [29], misdiagnosis is still problematic, as it is not commonly considered in differential diagnosis and due to the fact that its asymptomatic presentation masks the true incidence of duodenal diverticula. 

However, in the present study we found that the prevalence of duodenal diverticula is lower in South Indians, when compared to other studies. A vegetarian diet and high intake of fiber could be significantly associated with lower risk of diverticular diseases [30] for they were correlated with rapid bowel transit times [31, 32], thus reducing the intraluminal pressure. Small bowel diverticula, in fact can be found in patients older than 50 years with peristaltic disorders, such as progressive systemic sclerosis, visceral myopathy, and visceral neuropathies leading to an increase in intraluminal pressure [33]. Thus, a low fiber diet and advancing age might contribute to the risk of developing acquired duodenal diverticula. The low prevalence rate of duodenal diverticula in South Indians could be attributed to the Indian diet, which consists of rice, wheat, ragi, lentils, vegetables, yoghurt, and less animal protein. 

## 5. Conclusion 

Duodenal diverticula being asymptomatic in 90% of cases makes it difficult to ascertain the incidence and constitutes a diagnostic and therapeutic challenge due to their non-specific presentation. Thus, awareness of the frequency and location of duodenal diverticula is of great importance in the diagnosis and management of pancreaticobiliary diseases. In conclusion, our report serves to highlight the prevalence and anatomical location of duodenal diverticula in South Indians. The present study revealed a lower prevalence (4.2%) of duodenal diverticula comparable to that in the literature, and whether this could be attributed to the Indian diet pattern needs further study. 

## Figures and Tables

**Table 1:** Prevalence of duodenal diverticula in various studies.

Author	Number of cases	Incidence	Solitary	Multiple
Baldwin (1911) [18]	105	15 (13.3%)	13 (92.8%)	1 (7.2%)
Grant (1935) [14]	133	15 (11.2%)	11 (73.3%)	4 (26.7%)
Ackermann (1943) [19]	50	11 (22%)	8 (72.7%)	4 (36.3%)
Minoru and Atsuyoshi (1973) [8]	129	31.8%	—	—
Our study	120	5 (4.2%)	5 (100%)	—

**Table 2:** Distribution of diverticula in various parts of duodenum.

Author	No. of Diverticula	I	II	III	IV
Baldwin 1911 [9]	15	0	9	5	1
Case 1920 [2]	85	17	49	19	0
Spriggs and Marxer 1926 [26]	51 (38 cases)	1	30	16	4
Grant Boileau 1935 [14]	20 (15 cases)	0	14 (1) Jn. of I and II (2) Jn. of II and III	3 (from III and IV)	
Ackermann 1943 [19]	14 (11 cases)	0	5	5	3
Our study	5	0	3	2	0
